# Organ-Specific Phenolic Profiling and Integrated Antioxidant Evaluation of *Cicer isauricum* by LC–ESI–MS/MS and Multi-Assay Approach

**DOI:** 10.3390/ijms27135850

**Published:** 2026-06-29

**Authors:** Salih Akca, Bedrettin Selvi

**Affiliations:** Department of Biology, Faculty of Arts and Sciences, Tokat Gaziosmanpaşa University, 60250 Tokat, Türkiye; salih-akca-ankara-224@hotmail.com

**Keywords:** *Cicer isauricum*, antioxidant activity, phenolic compounds, LC–MS/MS, RACI

## Abstract

This study presents an integrated evaluation of the organ-specific phenolic composition and antioxidant activity of *Cicer isauricum*. Extracts obtained from leaves, stems, and roots were analyzed using liquid chromatography–electrospray ionization tandem mass spectrometry (LC–ESI–MS/MS) and multiple in vitro antioxidant assays. LC–MS/MS analysis revealed a distinct organ-dependent distribution of phenolic compounds. Stem extracts were characterized by high levels of hyperoside (2227.97 µg/g extract), luteolin (298.22 µg/g), and eriodictyol (434.03 µg/g), while leaves were rich in hyperoside (1162.42 µg/g), hesperidin (459.40 µg/g) and kaempferol (182.88 µg/g). Root extracts were dominated by flavan-3-ols, particularly (+)-catechin (355.93 µg/g) and (–)-epicatechin (59.58 µg/g), indicating a differentiated metabolic profile. Antioxidant assays, including cupric reducing antioxidant capacity (CUPRAC), ferric reducing antioxidant power (FRAP), 2,2-diphenyl-1-picrylhydrazyl (DPPH) radical scavenging, and 2,2′-azinobis(3-ethylbenzothiazoline-6-sulfonic acid) (ABTS) assays, demonstrated that root extracts exhibited the strongest activity, with the lowest IC_50_ values (DPPH: 4.15 mg/mL; ABTS: 1.41 mg/mL) and highest reducing power (FRAP EC_50_: 0.41 mg/mL; CUPRAC EC_50_: 1.78 mg/mL). Correlation analysis confirmed strong associations between total phenolic content and antioxidant capacity, while compound-level evaluation highlighted flavan-3-ols as major contributors. These findings identify roots of *C. isauricum* as a promising source of natural antioxidants.

## 1. Introduction

The increasing demand for natural bioactive compounds has stimulated extensive research on plant-derived secondary metabolites, particularly phenolic compounds, due to their strong antioxidant properties and potential health-promoting effects. The Fabaceae family represents one of the largest angiosperm families, comprising more than 19,000 species worldwide, and is well known for its rich phytochemical diversity, especially phenolic acids, flavonoids, and related metabolites [1,2]. These compounds play a crucial role in plant defense mechanisms and are widely associated with various biological activities, including antioxidant and protective effects against oxidative stress [3,4].

The genus *Cicer* (Fabaceae) includes both cultivated and wild species distributed mainly in the Mediterranean basin and Western Asia. While *Cicer arietinum* has been extensively investigated for its nutritional and functional properties, wild *Cicer* species remain relatively underexplored in terms of their phytochemical composition and biological activities [1,5]. Previous studies have consistently reported that *Cicer* species are rich in phenolic compounds, particularly flavonoids and phenolic acids, which are closely associated with their antioxidant properties [5,6,7].

Among these species, *Cicer isauricum* is an endemic taxon with limited geographical distribution, and comprehensive studies focusing on its phytochemical composition are still lacking. In particular, there is a notable absence of studies integrating detailed LC–MS/MS-based phenolic profiling with antioxidant activity evaluation across different anatomical parts of this species. This represents a significant gap in the literature and highlights the need for systematic investigation.

Extraction techniques play a crucial role in determining both the yield and composition of plant-derived bioactive compounds. Ultrasound-assisted extraction (UAE) has gained considerable attention as an efficient and environmentally friendly technique due to its ability to enhance mass transfer, reduce extraction time, and improve the recovery of phenolic compounds [8,9]. In addition, solvent polarity significantly affects extraction efficiency, and methanol has been widely reported as one of the most effective solvents for extracting a broad range of phenolic compounds from plant matrices [10,11].

Antioxidant activity is commonly evaluated using different in vitro assays such as DPPH, ABTS, FRAP, and CUPRAC, which reflect distinct mechanisms including radical scavenging and reducing power. The use of multiple complementary assays provides a more comprehensive and reliable evaluation of antioxidant potential [12,13].

Despite the increasing interest in the phytochemical composition and biological activities of leguminous plants, comprehensive studies focusing on endemic and underexplored *Cicer* species remain limited. In particular, *C. isauricum*, an endemic species distributed in Türkiye, has not been systematically investigated in terms of its organ-specific phenolic composition and antioxidant potential. Previous studies on *Cicer* species have predominantly focused on seeds, leaving vegetative organs such as leaves, stems, and roots largely unexplored, despite their potential as alternative sources of bioactive compounds.

In this context, the present study provides a detailed and integrative evaluation of the phenolic profile and antioxidant activity of different anatomical parts of *C. isauricum* using ultrasound-assisted methanolic extraction. The study combines quantitative phytochemical analysis by LC–ESI–MS/MS with multiple in vitro antioxidant assays reflecting different mechanisms, together with correlation-based interpretation and Relative Antioxidant Capacity Index (RACI) evaluation.

To our knowledge, this is among the first studies to comprehensively investigate the organ-dependent phenolic composition and mechanism-oriented antioxidant activity of *C. isauricum*. Beyond conventional antioxidant screening, this work establishes direct relationships between individual phenolic constituents and antioxidant mechanisms, thereby providing deeper insight into structure–activity interactions. Furthermore, the findings highlight the potential of non-edible plant organs, particularly roots, as rich and previously underutilized sources of natural antioxidants within the genus *Cicer*.

## 2. Results and Discussion

### 2.1. Total Phenolic and Flavonoid Contents

**Figure 1:**
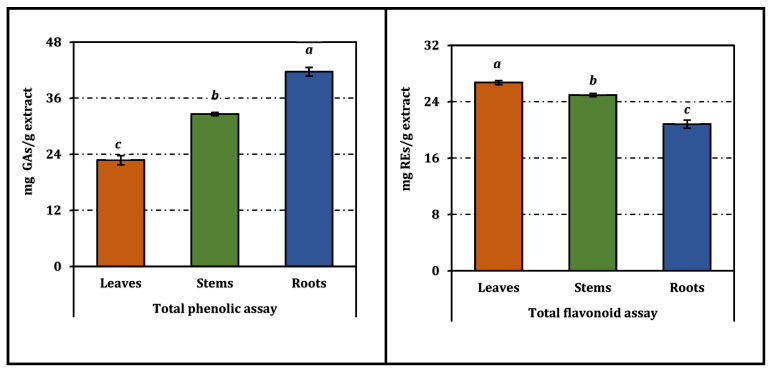
Total flavonoid and phenolic contents of *C. isauricum* extracts. REs and GAEs: Rutin and gallic acid equivalents, respectively. Values sharing the same superscript letters (a–c) on the bars are not significantly different according to Tukey’s HSD test (*p* < 0.05). All results are expressed as mean ± standard deviation (SD) of three parallel measurements (*n* = 3).

The total phenolic and flavonoid contents of different anatomical parts (leaves, stems, and roots) of *Cicer isauricum* extracts exhibited statistically significant variations (*p* < 0.05). The total phenolic content was the highest in the roots (41.65 ± 0.93 mg GAEs/g extract), followed by stems (32.62 ± 0.37 mg GAEs/g extract) and leaves (22.74 ± 1.00 mg GAEs/g extract). In contrast, total flavonoid content showed an opposite trend, being the highest in the leaves (26.73 ± 0.30 mg REs/g extract), while stems and roots contained lower amounts (Figure 1). These findings clearly indicate an organ-dependent distribution of phenolic compounds.

The higher accumulation of phenolic compounds in roots may be related to their protective role against soil-borne stress factors, including microbial attack and oxidative stress. Roots are known to serve as storage sites for phenolic acids and related metabolites involved in plant defense mechanisms [14,15]. In contrast, the elevated flavonoid content observed in leaves can be attributed to their involvement in UV protection and oxidative stress regulation, as flavonoids act as key photoprotective compounds in photosynthetically active tissues [16].

Although specific studies on *C. isauricum* are limited, similar organ-dependent trends have been reported in other *Cicer* species. For instance, in *C. arietinum*, phenolic compounds have been shown to accumulate at higher levels in certain plant tissues depending on their physiological roles, while flavonoids are predominantly found in aerial parts exposed to environmental stressors [1,6]. These findings are in agreement with the results obtained in the present study. In quantitative terms, the total phenolic content reported in the present study, particularly for root extracts (41.65 mg GAEs/g extract), is higher than or comparable to values reported for several leguminous plants, including *C. arietinum*, where TPC values are often below 30 mg GAEs/g extract [6,7]. This suggests that *C. isauricum* may represent a relatively richer source of phenolic compounds among *Cicer* species.

TPC values reported for legume species show considerable variability depending on seed type, cultivar, and growing conditions. For instance, lentils contain higher TPC (4.86–9.60 mg GAEs/g) compared to chickpeas (0.98 mg GAEs/g), peas (0.65–1.14 mg GAEs/g), and soybeans (1.57–5.57 mg GAEs/g) [6]. Similarly, significant variations have been reported among bean varieties (0.57–6.99 mg GAEs/g), with higher values generally observed in pigmented seeds. The phenolic content is also strongly associated with seed coat characteristics, where colored and darkening varieties may exhibit several-fold higher TPC compared to lighter counterparts [17,18]. These variations support the relatively high TPC observed in *C. isauricum*, particularly in root extracts.

Furthermore, the observed differences among plant organs may also be explained by tissue-specific biosynthesis and accumulation pathways of secondary metabolites. Phenolic acids are generally associated with structural and storage tissues such as roots, whereas flavonoids are actively synthesized in leaves due to their role in light absorption and reactive oxygen species scavenging [16,19]. This metabolic specialization supports the distribution pattern observed in *C. isauricum*.

These findings confirm a clear organ-dependent phytochemical distribution, with roots as the main source of phenolics and leaves enriched in flavonoids. This distribution pattern is consistent with previously reported findings in leguminous plants and supports the functional roles of these compounds in plant physiology [1,14,16].

### 2.2. LC–MS/MS Phenolic Profile

The LC–ESI–MS/MS analysis enabled the identification and quantification of a wide range of phenolic compounds in different anatomical parts (leaves, stems, and roots) of *C. isauricum*. A total of 28 phenolic compounds, including phenolic acids, flavonoids, and their derivatives, were detected, revealing a clear organ-dependent distribution pattern (Table 1). Only compounds that were positively identified and quantified using authenticated reference standards are presented in Table 1.

The stem extract was particularly rich in several phenolic compounds, showing the highest concentrations of hyperoside (2227.97 ± 5.54 µg/g extract), eriodictyol (434.03 ± 3.43 µg/g extract), luteolin (298.22 ± 0.81 µg/g extract), quercetin (198.41 ± 1.16 µg/g extract), and vanillin (130.54 ± 2.11 µg/g extract). In contrast, the leaf extract was characterized by high levels of hesperidin (459.40 ± 10.55 µg/g extract), kaempferol (182.88 ± 0.13 µg/g extract), luteolin (110.52 ± 0.32 µg/g extract), and hyperoside (1162.42 ± 30.08 µg/g extract). The root extract exhibited a distinct profile dominated by (+)-catechin (355.93 ± 1.23 µg/g extract), (−)-epicatechin (59.58 ± 0.38 µg/g extract), and apigenin 7-glucoside (109.34 ± 0.62 µg/g extract), indicating a higher accumulation of certain flavan-3-ols and glycosylated flavonoids in underground tissues.

In addition to flavonoids, several phenolic acids such as protocatechuic acid (85.58 ± 0.41 µg/g extract), syringic acid (73.21 ± 0.51 µg/g extract), and ferulic acid (55.25 ± 0.52 µg/g extract) were predominantly found in the stem extract, suggesting that stems serve as an important reservoir for both phenolic acids and flavonoid derivatives. This distribution pattern is consistent with the total phenolic content results, where stems and roots exhibited higher phenolic accumulation compared to leaves.

The predominance of phenolic acids and certain flavonoids in stems and roots may be associated with their structural and protective roles in plant physiology. Phenolic acids are known to contribute to cell wall strengthening and defense against pathogens, particularly in supporting tissues [14,15]. On the other hand, the high abundance of flavonoids such as kaempferol, luteolin, and hesperidin in leaves can be explained by their involvement in UV protection and oxidative stress mitigation in photosynthetically active tissues [16].

Although LC–MS/MS-based studies on *C. isauricum* are scarce, similar phenolic profiles have been reported in other *Cicer* species. In *C. arietinum*, flavonoids such as quercetin, kaempferol, and their glycosides, along with phenolic acids including caffeic and ferulic acids, have been identified as major constituents [1,6]. Moreover, previous LC–MS/MS studies on plant phenolics have consistently reported flavonoid glycosides, including rutin-type compounds, together with phenolic acids as dominant constituents [20], which is in agreement with the present findings. Observed differences in phenolic composition among plant organs are likely driven by tissue-specific biosynthesis and metabolite accumulation, shaped by physiological function and environmental factors, leading to distinct phytochemical profiles [16,19].

Overall, the LC–MS/MS results demonstrate that *C. isauricum* possesses a rich and organ-specific phenolic composition, with stems showing the highest diversity and concentration of phenolic compounds, leaves being enriched in flavonoids, and roots characterized by specific flavan-3-ol accumulation. This distribution pattern aligns with previously reported findings in leguminous species and supports the functional specialization of phenolic compounds in different plant organs [1,14,16].

### 2.3. Antioxidant Activity

**Figure 2:**
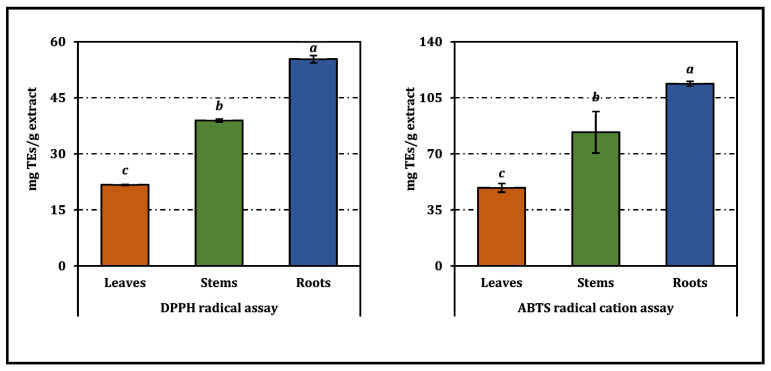

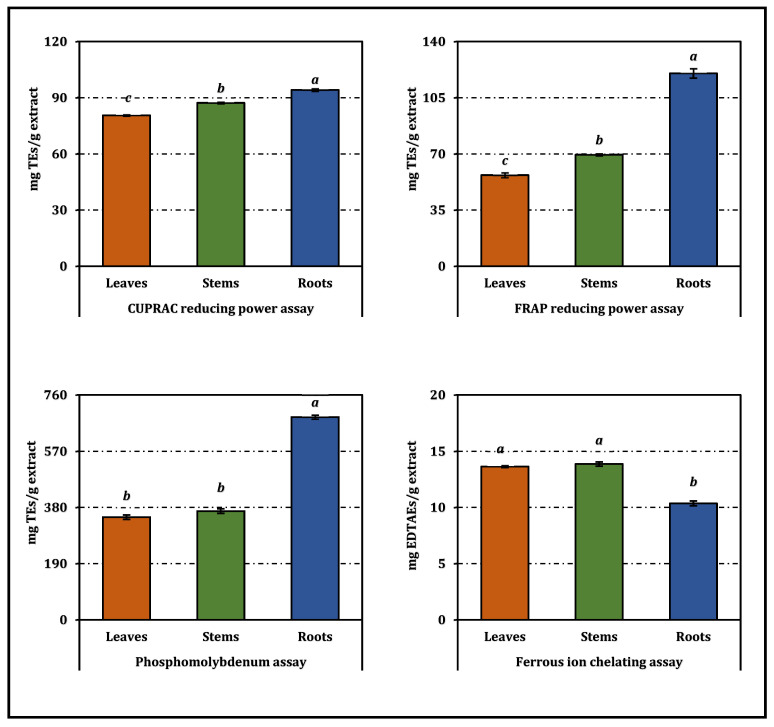
Antioxidant activity of *C. isauricum* extracts. TEs and EDTAEs, trolox and ethylenediaminetetraacetic acid (disodium salt) equivalents, respectively. Values sharing the same superscript letters (a–c) on the bars are not significantly different according to Tukey’s HSD test (*p* < 0.05). All results are expressed as mean ± standard deviation (SD) of three parallel measurements (*n* = 3).

The antioxidant activities of the methanolic extracts obtained from the leaves, stems, and roots of *Cicer isauricum* were evaluated using complementary in vitro assays reflecting different mechanisms, including radical scavenging, reducing power, total antioxidant capacity, and metal chelation. The results were expressed both as IC_50_/EC_50_ values (Table 2) and as standard equivalents (Figure 2), providing a comprehensive evaluation of antioxidant potential. The overall antioxidant profile was organ-dependent, although the magnitude of the differences varied according to the assay.

Among the tested organs, the root extract generally exhibited the strongest antioxidant performance in radical scavenging and reducing power assays. It showed the lowest IC_50_ values in DPPH (4.15 ± 0.07 mg/mL) and ABTS (1.41 ± 0.02 mg/mL), compared to stems (DPPH: 5.91 ± 0.06 mg/mL; ABTS: 1.95 ± 0.31 mg/mL) and leaves (DPPH: 10.59 ± 0.07 mg/mL; ABTS: 3.31 ± 0.19 mg/mL). This trend was consistent with Trolox equivalent values, where the root extract displayed the highest activity (DPPH: 55.37 ± 0.98 mg TEs/g extract; ABTS: 113.83 ± 1.56 mg TEs/g extract), followed by stems and leaves.

Similar results were obtained in reducing power assays. The root extract exhibited the lowest EC_50_ values in CUPRAC (1.78 ± 0.01 mg/mL) and FRAP (0.41 ± 0.01 mg/mL), while stems (CUPRAC: 1.92 ± 0.01 mg/mL; FRAP: 0.70 ± 0.01 mg/mL) and leaves (CUPRAC: 2.08 ± 0.01 mg/mL; FRAP: 0.86 ± 0.02 mg/mL) showed lower reducing capacity. Correspondingly, the highest reducing power values were observed in roots (CUPRAC: 94.18 ± 0.68 mg TEs/g extract; FRAP: 120.17 ± 2.98 mg TEs/g extract).

In the phosphomolybdenum assay, the root extract again exhibited the highest total antioxidant capacity (685.22 ± 6.90 mg TEs/g extract; EC_50_ = 0.57 ± 0.01 mg/mL), whereas stems (367.83 ± 7.50 mg TEs/g extract) and leaves (347.25 ± 7.20 mg TEs/g extract) showed lower values. In contrast, metal chelating activity followed a different pattern, with stems (IC_50_ = 1.05 ± 0.01 mg/mL) and leaves (IC_50_ = 1.07 ± 0.01 mg/mL) exhibiting stronger activity than roots (IC_50_ = 1.41 ± 0.03 mg/mL).

These results are in line with previous studies indicating that antioxidant activity in plant extracts is more closely associated with total phenolic content than with total flavonoid content alone [6,13]. In the present study, the root extract had the highest total phenolic content, whereas leaves had the highest flavonoid content, supporting the observed activity distribution.

In addition to *Cicer* species, antioxidant activities reported for other legumes further support the variability in phenolic-driven bioactivity. For instance, the DPPH radical scavenging activity of horse gram flour extract (IC_50_ = 22.9 μg/mL) was reported to be higher than that of chickpea (IC_50_ = 31.4 μg/mL) and cowpea (IC_50_ = 48.7 μg/mL) extracts [21]. Similarly, studies on peanut (*Arachis hypogaea*) demonstrated that antioxidant capacity varies markedly among different seed fractions, with DPPH values ranging from 1.50 to 28.9 μmol Trolox equivalents (TE)/g and FRAP values between 2.04 and 1111.3 μmol Fe^2+^/g depending on the plant part [22].

However, it should be noted that direct quantitative comparison between these studies and the present results is not appropriate due to differences in expression units (e.g., IC_50_ in mg/mL vs μg/mL, or TE/Fe^2+^ equivalents), extraction procedures, and plant matrices. Nevertheless, these reports collectively highlight that antioxidant activity in legumes is highly dependent on both plant part and phenolic composition, which is in agreement with the organ-dependent antioxidant behavior observed for *C. isauricum* in this study.

Similarly, studies on *C. reticulatum*, the wild progenitor of chickpea, have shown that antioxidant capacity is strongly influenced by phenolic composition and environmental factors, with phenolic-rich extracts exhibiting enhanced activity [23]. The high antioxidant performance observed in the root extract of *C. isauricum* is therefore consistent with the general trend reported for wild legumes, where stress-adapted species tend to accumulate higher levels of bioactive phenolics.

Furthermore, it has been reported that in *Cicer* species, seed-based extracts are the most extensively studied matrices, whereas investigations focusing on vegetative organs remain limited. Therefore, the present study provides novel insights by demonstrating that non-edible plant parts, particularly roots, may represent a significant source of natural antioxidants within the genus. Comparable findings have recently been reported for *Alkanna tubulosa* and *Picnomon acarna*, where phenolic-rich extracts characterized by LC–ESI–MS/MS exhibited strong radical scavenging and reducing power activities, further supporting the close relationship between phenolic composition and antioxidant performance [24,25].

At the compound level, LC–MS/MS analysis revealed that the root extract was particularly rich in catechin and epicatechin derivatives, whereas stems contained higher levels of flavonoids such as hyperoside, luteolin, quercetin, and eriodictyol. The antioxidant activity of these compounds is closely related to their structural features. Flavan-3-ols such as catechin and epicatechin exhibit strong antioxidant capacity due to their multiple hydroxyl groups and catechol-type B-ring structure, which enhance electron-donating ability and radical stabilization. Similarly, flavonoids such as quercetin and luteolin possess structural elements, including a 2,3-double bond conjugated with a 4-oxo function and ortho-dihydroxyl groups in the B-ring, which are known to increase radical scavenging and reducing power [4,16,26]. In contrast, glycosylated flavonoids (e.g., hyperoside, hesperidin, apigenin 7-glucoside) generally exhibit lower antioxidant activity compared to their aglycone forms due to steric hindrance and reduced availability of free hydroxyl groups [3,4]. This may explain why stem extracts, despite being rich in flavonoid glycosides, showed comparatively lower activity than root extracts dominated by flavan-3-ols.

Furthermore, phenolic acids such as ferulic, syringic, and caffeic acids contribute differently depending on their substitution patterns, with hydroxyl and methoxy groups influencing both electron transfer and metal chelation capacity [12,26]. These structural differences collectively explain the assay-dependent antioxidant responses observed in this study.

**Figure 3:**
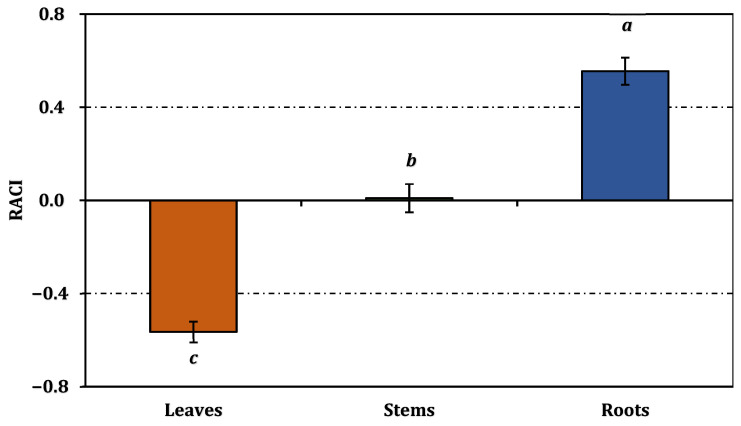
Relative antioxidant capacity index of *C. isauricum* extracts. Values sharing the same superscript letters (a–c) on the bars are not significantly different according to Tukey’s HSD test (*p* < 0.05).

In order to provide an integrative evaluation of antioxidant performance across different assay systems, the Relative Antioxidant Capacity Index (RACI) was calculated (Figure 3). The RACI values clearly reflected the overall antioxidant potential of the extracts, with roots showing the highest value (0.56), followed by stems (0.01) and leaves (−0.56). This ranking was fully consistent with the results obtained from individual antioxidant assays, where the root extract exhibited superior radical scavenging activity and reducing power. The positive RACI value of the root extract indicates an above-average antioxidant capacity, whereas the negative value observed for leaves suggests relatively lower overall activity, while the near-zero value of stems reflects an intermediate antioxidant potential. The agreement between RACI scores and individual assay results confirms the robustness of the antioxidant evaluation and highlights the advantage of using RACI as an integrative approach to combine data obtained from different analytical methods with varying measurement scales [27]. Given the limited number of data points (*n* = 3), these correlation coefficients may be overestimated and should therefore be interpreted as indicative rather than definitive relationships. This integrative interpretation further supports the observed organ-dependent antioxidant behavior and provides a reliable basis for correlation-based analyses.

Pearson correlation analysis provided further insight into these relationships (Table 3). Strong positive correlations were observed among radical scavenging and reducing power assays, with DPPH correlating strongly with CUPRAC (r = 0.998) and ABTS (r = 0.981), and FRAP correlating with phosphomolybdenum (r = 0.989). In contrast, metal chelating activity showed strong negative correlations with these assays (e.g., FRAP: r = −0.964), confirming mechanistic differences. Total phenolic content showed strong positive correlations with DPPH (r = 0.996), ABTS (r = 0.984), CUPRAC (r = 0.993), and FRAP (r = 0.936), whereas total flavonoids showed inverse relationships but were positively correlated with metal chelation (r = 0.931). At the individual compound level, catechin and apigenin 7-glucoside showed near-perfect correlations with phosphomolybdenum (r = 0.999), while epicatechin strongly correlated with FRAP (r = 0.997), highlighting their major contribution to antioxidant activity. These correlation patterns generally support the proposed structure–activity relationships between phenolic composition and antioxidant mechanisms. The very strong positive correlations of catechin and epicatechin with reducing power assays (FRAP and CUPRAC) can be attributed to their high electron-donating capacity arising from multiple hydroxyl substitutions, which enhance redox potential and radical stabilization [4,26]. Likewise, the strong association between apigenin 7-glucoside and total antioxidant capacity suggests that glycosylated flavonoids may contribute more effectively to certain mechanisms such as phosphomolybdenum-based total antioxidant activity rather than direct radical scavenging [12].

Conversely, the negative correlations observed for several phenolic acids and flavonoid glycosides indicate that not all phenolic compounds contribute equally to antioxidant performance, reinforcing the importance of structural features in determining activity [3]. Overall, these findings confirm that antioxidant behavior is governed not only by total phenolic content but also by the molecular structure of individual constituents.

Overall, the antioxidant activity of *C. isauricum* is strongly dependent on plant organ and is governed not only by total phenolic content but also by the composition and distribution of individual compounds. Radical scavenging and reducing power are primarily associated with flavan-3-ols, whereas metal chelation is influenced by different compound groups, emphasizing the importance of mechanism-specific evaluation. To the best of our knowledge, this is one of the first comprehensive reports evaluating the antioxidant activity of different anatomical parts of *C. isauricum* in relation to their detailed phenolic composition and correlation-based mechanistic interpretation, thereby providing new insights into the bioactive potential of this endemic species.

## 3. Materials and Methods

### 3.1. Plant Material

The plant *C. isauricum* P.H. Davis was collected on 22 August 2025, from Gidefi Mountain (36°53′53″ N, 31°54′47″ E) at an altitude of 1525 m in Pınarbaşı village, Akseki district, Antalya province, Türkiye. The species was collected by Salih Akça, identified by Dr. Bedrettin Selvi, and a specimen with herbarium number (GOPU 8592) was deposited in the Herbarium of the Faculty of Science and Literature, Gaziosmanpaşa University, Tokat. In this study, three different parts of the plant were used: leaves, stems, and roots. These parts were collected separately during the harvesting process. After collection, the plant material was air-dried in a shaded environment for several weeks. The dried plant parts were then ground into a fine powder using a laboratory mill and used for further analysis. The powdered plant materials were transferred into airtight containers and stored at 4 °C in the dark until extraction.

### 3.2. Methanol Extraction

All solvents, reagents, and reference standards used in this study were of analytical or LC–MS grade. Methanol (HPLC grade) and the phenolic reference standards (Supplementary Materials), together with the Folin–Ciocalteu reagent, aluminium chloride, rutin, DPPH, ABTS, Trolox, and EDTA (disodium salt), were purchased from Sigma-Aldrich (St. Louis, MO, USA) and Fluka (St. Louis, MO, USA), except for verbascoside, protocatechuic acid, and hyperoside, which were obtained from HWI Analytik (Ruelzheim, Germany). Formic acid (LC–MS grade) was purchased from Merck (Darmstadt, Germany), and ultra-pure water was produced using a Milli-Q Plus system (Millipore, Bedford, MA, USA).

Methanol extraction of the plant materials (leaves, stems, and roots) was performed using an ultrasound-assisted extraction (UAE) approach. For each organ, 5 g of material was extracted with 100 mL of HPLC-grade methanol (1:20, *w*/*v*) in an Elma ultrasonic bath (Elma Schmidbauer GmbH, Singen, Germany) operating at 40 kHz and 260 W at approximately 30 °C for 60 min. [28,29]. Following extraction, the mixtures were filtered through Whatman No. 1 filter paper (Whatman, Maidstone, UK) and the solvent was removed under reduced pressure using a Heidolph rotary evaporator (Heidolph Instruments, Schwabach, Germany). The resulting extracts were concentrated and stored at 4 °C until further analyses. The extraction yields were calculated based on the dry weight of the plant material and determined as 6.43%, 3.07%, and 4.55% for leaves, stems, and roots, respectively (*w*/*w*).

### 3.3. Determination of the Phenolic Compositions of the Extracts

The total phenolic content of the extracts was determined using the Folin–Ciocalteu assay and expressed as gallic acid equivalents (GAEs), whereas total flavonoid content was measured by the aluminium chloride colorimetric method and reported as rutin equivalents (REs) [30,31].

The phenolic composition of the extracts was characterized using a validated liquid chromatography–electrospray ionization tandem mass spectrometry (LC–ESI–MS/MS) method, enabling the simultaneous determination of multiple phenolic compounds [32]. Quantitative analyses were performed on an Agilent Technologies 1260 Infinity liquid chromatography system coupled with a 6420 Triple Quadrupole mass spectrometer (Agilent Technologies, Santa Clara, CA, USA).

Chromatographic separation was achieved using a Poroshell 120 EC-C18 column (100 mm × 4.6 mm, 2.7 μm particle size; Agilent Technologies, Santa Clara, CA, USA) with a binary mobile phase consisting of solvent A (0.1% formic acid in water) and solvent B (methanol), under gradient elution conditions. The column temperature was maintained at 25 °C, the flow rate was set at 0.4 mL min^−1^, and the injection volume was 2 μL.

Mass spectrometric detection was performed using an electrospray ionization (ESI) source operating in both positive and negative ion modes under multiple reaction monitoring (MRM) conditions. Identification and quantification of phenolic compounds were carried out by comparing retention times and characteristic ion transitions with those of authentic reference standards.

Optimized mass spectrometric parameters, including capillary voltage, gas temperature, nebulizer pressure, and compound-specific collision energies, as well as method validation data, are provided in Tables S1 and S2 (Supplementary Materials). Representative LC–ESI–MS/MS chromatograms of leaf, stem, and root extracts together with numbered peak assignments are provided in Supplementary Figure S1.

### 3.4. Biological Activity

All absorbance measurements (Section 3.3 and Section 3.4) were recorded using a Shimadzu UV-Vis spectrophotometer (Shimadzu Corporation, Kyoto, Japan).

The antioxidant capacity of the extracts was evaluated using multiple in vitro assays based on different mechanisms of action. Total antioxidant capacity was determined by the phosphomolybdenum method [33]. Radical scavenging activity was assessed using the DPPH assay [34] and the ABTS assay [35]. Metal chelating activity on ferrous ions was determined according to the method of Dinis et al. [36]. The reducing power of the extracts was evaluated using the CUPRAC method [37] and the FRAP assay [38]. All assays were performed with slight modifications according to previously reported procedures [24,25,28].

Results were expressed as IC_50_ or EC_50_ values and as standard equivalents (e.g., Trolox and EDTA equivalents). Detailed experimental procedures and assay conditions are provided in the Supplementary Materials.

### 3.5. Statistical Analysis

All experimental data are expressed as mean ± standard deviation (SD) of three parallel measurements (*n* = 3), representing technical replicates obtained from the same extract. Statistical analyses were performed using SPSS version 26.0 (IBM Corp., Armonk, NY, USA). Differences among samples were evaluated by one-way analysis of variance (ANOVA), followed by Tukey’s post hoc test, with *p* < 0.05 considered statistically significant.

Pearson’s correlation analysis was applied to assess the relationships between phenolic composition and antioxidant activity parameters. Correlation coefficients (r) and corresponding *p*-values were calculated to determine the strength and significance of these associations.

To enable a comprehensive comparison of antioxidant activity results obtained from different assay systems, the Relative Antioxidant Capacity Index (RACI) was calculated according to Sun and Tanumihardjo [27]. RACI values were obtained by standardizing the results of each assay using z-score transformation, allowing the integration of data with different measurement scales into a unified index.

It should be noted that the results are based on technical replicates (*n* = 3); therefore, the findings should be interpreted with caution, as they may not fully reflect biological variability.

### 3.6. Use of Artificial Intelligence

Artificial intelligence (AI) tools were used solely for language editing and improvement of clarity. AI had no role in the study design, data collection, analysis, interpretation, or conclusions of this work. All content was critically reviewed and approved by the authors.

## 4. Conclusions

This study provides a comprehensive evaluation of the organ-specific phytochemical composition and antioxidant activity of *C. isauricum*. Antioxidant capacity was strongly dependent on plant organ and closely associated with total phenolic content, particularly flavan-3-ols such as catechin and epicatechin.

The integrative use of LC–ESI–MS/MS, multi-assay antioxidant testing, correlation analysis, and RACI enabled a mechanism-oriented interpretation, showing that radical scavenging and reducing power are driven by electron-donating phenolics, while metal chelation involves different compound groups. These findings provide supportive evidence for structure–activity relationships, highlighting the key role of hydroxylation, conjugation, and glycosylation in antioxidant efficiency. Notably, roots emerged as a rich and underutilized source of bioactive compounds.

Overall, this study represents the first comprehensive report on the organ-specific antioxidant profile of *C. isauricum* and underscores the potential of endemic Cicer species as valuable sources of natural antioxidants.

## Figures and Tables

**Table 1:** Quantitative distribution of phenolic compounds identified in *C. isauricum* extracts (µg/g extract).

Class	Compound	Leaves	Stems	Roots
Hydroxybenzoic acids	Gallic acid	3.10 ± 0.03 ^b^	9.92 ± 0.10 ^a^	3.23 ± 0.17 ^b^
	Protocatechuic acid	28.72 ± 0.15 ^b^	85.58 ± 0.41 ^a^	30.16 ± 0.48 ^b^
	3-Hydroxybenzoic acid	35.37 ± 1.35 ^b^	44.05 ± 0.35 ^a^	34.69 ± 0.69 ^b^
	4-Hydroxybenzoic acid	56.05 ± 0.90 ^b^	75.03 ± 1.56 ^a^	57.43 ± 0.94 ^b^
	Syringic acid	22.81 ± 0.29 ^c^	73.21 ± 0.51 ^a^	36.29 ± 0.10 ^b^
Hydroxyphenylacetic acids	3,4-Dihydroxyphenylacetic acid	4.74 ± 0.37 ^b^	6.57 ± 0.15 ^a^	4.18 ± 0.03 ^b^
Hydroxycinnamic acids	Chlorogenic acid	3.68 ± 0.01 ^b^	8.67 ± 0.16 ^a^	2.84 ± 0.14 ^c^
	Caffeic acid	4.48 ± 0.14 ^b^	9.05 ± 0.09 ^a^	1.59 ± 0.01 ^c^
	Sinapic acid	16.10 ± 0.15 ^a^	14.32 ± 0.59 ^b^	7.48 ± 0.27 ^c^
	p-Coumaric acid	29.34 ± 0.68 ^b^	49.62 ± 0.75 ^a^	8.99 ± 0.26 ^c^
	Ferulic acid	45.74 ± 0.10 ^b^	55.25 ± 0.52 ^a^	6.63 ± 0.06 ^c^
	Rosmarinic acid	4.61 ± 0.03 ^b^	9.31 ± 0.41 ^a^	4.87 ± 0.10 ^b^
	2-Hydroxycinnamic acid	nd	nd	nd
Flavan-3-ols	(+)-Catechin	6.35 ± 0.13 ^c^	13.80 ± 0.45 ^b^	355.93 ± 1.23 ^a^
	(−)-Epicatechin	2.11 ± 0.01 ^c^	10.08 ± 0.78 ^b^	59.58 ± 0.38 ^a^
Flavanonols	Taxifolin	8.16 ± 0.23 ^c^	84.04 ± 0.99 ^a^	20.95 ± 0.56 ^b^
Flavanones	Eriodictyol	58.02 ± 0.14 ^b^	434.03 ± 3.43 ^a^	30.93 ± 0.09 ^c^
Flavanone glycosides	Hesperidin	459.40 ± 10.55 ^a^	127.51 ± 1.03 ^b^	nd
Flavonol glycosides	Hyperoside	1162.42 ± 30.08 ^b^	2227.97 ± 5.54 ^a^	43.04 ± 0.30 ^c^
Flavonols	Quercetin	57.34 ± 0.31 ^b^	198.41 ± 1.16 ^a^	13.55 ± 0.38 ^c^
	Kaempferol	182.88 ± 0.13 ^a^	150.09 ± 2.32 ^b^	33.64 ± 0.60 ^c^
Flavone glycosides	Luteolin 7-glucoside	59.61 ± 0.12 ^b^	97.09 ± 1.68 ^a^	10.10 ± 0.15 ^c^
	Apigenin 7-glucoside	7.41 ± 0.48 ^b^	8.97 ± 0.74 ^b^	109.34 ± 0.62 ^a^
Flavones	Luteolin	110.52 ± 0.32 ^b^	298.22 ± 0.81 ^a^	18.18 ± 0.06 ^c^
	Apigenin	24.48 ± 0.33 ^b^	63.55 ± 0.70 ^a^	10.81 ± 0.01 ^c^
Phenylethanoid glycosides	Verbascoside	5.43 ± 0.05 ^b^	10.19 ± 0.14 ^a^	5.11 ± 0.01 ^b^
Phenolic aldehydes	Vanillin	40.71 ± 0.37 ^b^	130.54 ± 2.11 ^a^	43.62 ± 0.25 ^b^
Lignans	Pinoresinol	32.19 ± 0.51 ^a^	15.83 ± 0.42 ^b^	8.76 ± 0.25 ^c^

Compounds are grouped according to their major phenolic subclasses to facilitate interpretation of the organ-specific phenolic profile. Values sharing the same superscript letters (a–c) within the same row are not significantly different according to Tukey’s HSD test (*p* < 0.05). All results are expressed as mean ± standard deviation (SD) of three parallel measurements (*n* = 3). nd: Not detected.

**Table 2:** Antioxidant activities of *C. isauricum* extracts.

Assays	Leaves	Stems	Roots	Trolox	EDTA
Phosphomolybdenum (EC_50_: mg/mL)	1.12 ± 0.02 ^c^	1.06 ± 0.02 ^c^	0.57 ± 0.01 ^b^	0.39 ± 0.02 ^a^	-
CUPRAC reducing power (EC_50_: mg/mL)	2.08 ± 0.01 ^d^	1.92 ± 0.01 ^c^	1.78 ± 0.01 ^b^	0.17 ± 0.01 ^a^	-
FRAP reducing power (EC_50_: mg/mL)	0.86 ± 0.02 ^d^	0.70 ± 0.01 ^c^	0.41 ± 0.01 ^b^	0.049 ± 0.001 ^a^	-
DPPH radical (IC_50_: mg/mL)	10.59 ± 0.07 ^d^	5.91 ± 0.06 ^c^	4.15 ± 0.07 ^b^	0.23 ± 0.01 ^a^	-
ABTS radical cation (IC_50_: mg/mL)	3.31 ± 0.19 ^c^	1.95 ± 0.31 ^b^	1.41 ± 0.02 ^b^	0.16 ± 0.003 ^a^	-
Ferrous ion chelating (IC_50_: mg/mL)	1.07 ± 0.01 ^b^	1.05 ± 0.01 ^b^	1.41 ± 0.03 ^c^	-	0.015 ± 0.0003 ^a^

EDTAE mean ethylenediaminetetraacetic acid (disodium salt) equivalents. Values sharing the same superscript letters (a–d) within the same row are not significantly different according to Tukey’s HSD test (*p* < 0.05). All results are expressed as mean ± standard deviation (SD) of three parallel measurements (*n* = 3).

**Table 3:** Correlations among phenolic compounds and assays.

	TAP	DPPH	ABTS	CUPRAC	FRAP	FICA
DPPH	0.884					
ABTS	0.859	0.981				
CUPRAC	0.891	0.998	0.970			
FRAP	0.989	0.938	0.915	0.943		
FICA	−0.990	−0.828	−0.805	−0.838	−0.964	
Total flavonoid	−0.962	−0.966	−0.927	−0.972	−0.981	0.931
Total phenolic	0.877	0.996	0.984	0.993	0.936	−0.817
Gallic acid	−0.437	0.031	0.056	0.010	−0.311	0.530
Protocatechuic acid	−0.432	0.036	0.061	0.014	−0.304	0.528
3,4-Dihydroxyphenylacetic acid	−0.636	−0.208	−0.186	−0.222	−0.520	0.709
(+)-Catechin	0.999	0.868	0.839	0.877	0.984	−0.994
Chlorogenic acid	−0.567	−0.119	−0.089	−0.141	−0.450	0.650
3-Hydroxybenzoic acid	−0.503	−0.050	−0.039	−0.072	−0.389	0.594
4-Hydroxybenzoic acid	−0.390	0.080	0.113	0.055	−0.265	0.484
(−)-Epicatechin	0.997	0.917	0.891	0.923	0.997	−0.980
Caffeic acid	−0.760	−0.372	−0.340	−0.393	−0.664	0.825
Syringic acid	−0.207	0.271	0.287	0.250	−0.071	0.313
Vanillin	−0.427	0.042	0.062	0.021	−0.299	0.524
Verbascoside	−0.501	−0.043	−0.013	−0.065	−0.378	0.592
Taxifolin	−0.306	0.170	0.189	0.149	−0.173	0.410
Sinapic acid	−0.989	−0.940	−0.925	−0.941	−0.998	0.964
p-Coumaric acid	−0.838	−0.489	−0.462	−0.506	−0.756	0.891
Ferulic acid	−0.971	−0.749	−0.716	−0.763	−0.929	0.988
Luteolin 7-glucoside	−0.877	−0.555	−0.520	−0.573	−0.804	0.922
Hesperidin	−0.751	−0.971	−0.957	−0.963	−0.833	0.671
Hyperoside	−0.845	−0.500	−0.468	−0.518	−0.763	0.896
Rosmarinic acid	−0.405	0.065	0.100	0.040	−0.278	0.498
Apigenin 7-glucoside	0.999	0.865	0.834	0.874	0.983	−0.994
Pinoresinol	−0.768	−0.977	−0.964	−0.970	−0.846	0.691
Eriodictyol	−0.505	−0.047	−0.023	−0.068	−0.382	0.596
Quercetin	−0.642	−0.213	−0.186	−0.234	−0.531	0.722
Kaempferol	−0.987	−0.946	−0.916	−0.952	−0.998	0.961
Luteolin	−0.716	−0.311	−0.281	−0.330	−0.614	0.787
Apigenin	−0.661	−0.237	−0.210	−0.257	−0.551	0.738

Data show the Pearson Correlation Coefficients between the parameters. TAP: total antioxidant activity by phosphomolybdenum method. ABTS and DPPH: ABTS and DPPH radical scavenging activities, respectively. CUPRAC and FRAP: CUPRAC and FRAP reducing power potential; respectively. FICA: Ferrous ion chelating activity.

## Data Availability

The data generated and/or analysed during this study are available from the corresponding author on reasonable request.

## Supplementary Materials

The following supporting information can be downloaded at: https://www.mdpi.com/article/10.3390/ijms27135850/s1.
